# PEDF peptide plus hyaluronic acid stimulates cartilage regeneration in osteoarthritis via STAT3-mediated chondrogenesis

**DOI:** 10.1302/2046-3758.134.BJR-2023-0179.R2

**Published:** 2024-04-01

**Authors:** Yung-Chang Lu, Tsung-Chuan Ho, Chang-Hung Huang, Shu-I Yeh, Show-Li Chen, Yeou-Ping Tsao

**Affiliations:** 1 Department of Medical Research, Mackay Memorial Hospital, New Taipei City, Taiwan; 2 Department of Medicine, Mackay Medical College, New Taipei City, Taiwan; 3 School of Dentistry, National Yang Ming Chiao Tung University, Taipei, Taiwan; 4 Graduate Institute of Microbiology, College of Medicine, National Taiwan University, Taipei, Taiwan

**Keywords:** mesenchymal stem/stromal cells, monoiodoacetate, synovial inflammation, hyaluronic acid (HA), Osteoarthritic changes, peptide, cartilage regeneration, chondrogenesis, Intra-articular injection, Rats, chondrocytes, knees

## Abstract

**Aims:**

Pigment epithelium-derived factor (PEDF) is known to induce several types of tissue regeneration by activating tissue-specific stem cells. Here, we investigated the therapeutic potential of PEDF 29-mer peptide in the damaged articular cartilage (AC) in rat osteoarthritis (OA).

**Methods:**

Mesenchymal stem/stromal cells (MSCs) were isolated from rat bone marrow (BM) and used to evaluate the impact of 29-mer on chondrogenic differentiation of BM-MSCs in culture. Knee OA was induced in rats by a single intra-articular injection of monosodium iodoacetate (MIA) in the right knees (set to day 0). The 29-mer dissolved in 5% hyaluronic acid (HA) was intra-articularly injected into right knees at day 8 and 12 after MIA injection. Subsequently, the therapeutic effect of the 29-mer/HA on OA was evaluated by the Osteoarthritis Research Society International (OARSI) histopathological scoring system and changes in hind paw weight distribution, respectively. The regeneration of chondrocytes in damaged AC was detected by dual-immunostaining of 5-bromo-2'-deoxyuridine (BrdU) and chondrogenic markers.

**Results:**

The 29-mer promoted expansion and chondrogenic differentiation of BM-MSCs cultured in different defined media. MIA injection caused chondrocyte death throughout the AC, with cartilage degeneration thereafter. The 29-mer/HA treatment induced extensive chondrocyte regeneration in the damaged AC and suppressed MIA-induced synovitis, accompanied by the recovery of cartilage matrix. Pharmacological inhibitors of PEDF receptor (PEDFR) and signal transducer and activator of transcription 3 (STAT3) signalling substantially blocked the chondrogenic promoting activity of 29-mer on the cultured BM-MSCs and injured AC.

**Conclusion:**

The 29-mer/HA formulation effectively induces chondrocyte regeneration and formation of cartilage matrix in the damaged AC.

Cite this article: *Bone Joint Res* 2024;13(4):137–148.

## Article focus

- This study investigated the therapeutic effect of pigment epithelium-derived factor (PEDF) 29-mer on rat osteoarthritis (OA) and underlying mechanisms.

## Key messages

- The 29-mer improves the chondrogenic differentiation of bone marrow mesenchymal stem/stromal cells (BM-MSCs) in a defined culture medium in the presence of transforming growth factor (TGF)-β.
- Intra-articular injection of the 29-mer/hyaluronic acid (HA) induces the regeneration of chondrocytes and restores the cartilage matrix in the damaged articular cartilage (AC) induced by monosodium iodoacetate (MIA).

## Strengths and limitations

- Our findings highlight the intra-articular injection of the 29-mer/HA as a simple and effective way to improve OA therapy.
- The main limitation of this study is the inadequate identification of the stem cells responsible for cartilage regeneration, although BM-MSCs are suggested.

## Introduction

Osteoarthritis (OA), the most common joint disease, results from the breakdown of articular cartilage (AC) in synovial joints.^1,2^ With age, AC degenerates at a cellular level (i.e. chondrocytes) and the amount of cartilage matrix decreases, leading to an overall loss of hyaline cartilage height.^1,3^ AC has a limited ability to self-heal after trauma because of its avascular nature and the resting state of articular chondrocytes.^1^ The pharmacological treatment options for chondral defects are very limited; these include analgesics and anti-inflammatory drugs.^2^ In addition, autologous stem cell transplantation using mesenchymal stem/stromal cells (MSCs) from various tissue sources has shown potential in improving pain relief and AC regeneration in clinical OA studies.^3^

Adult MSCs are able to expand in vitro and therefore provide an attractive option for the preparation of autologous chondrocytes for delivery in OA therapy.^4,5^ However, high-quality cell therapy for OA treatment by MSCs is technique- and resource-intensive. In addition, chondrogenic markers, including transcription factor SRY-type high mobility group box 9 (Sox9) and cartilage matrix proteins including aggrecan and collagen type 2 (Col2), were found to be reduced during the chondrogenic differentiation of MSCs, leading to the development of hypertrophic cartilage, instead of hyaline cartilage, in culture and in vivo.^5-7^ Alternatively, stem cells present in the bone marrow (BM), synovium, perichondrium, adipose tissue, tendons, and superficial zone of the AC have been proposed as potential cell sources to heal chondral injuries.^8-12^

Pigment epithelium-derived factor (PEDF) is a multifunctional glycoprotein and has promising potential as a regenerative therapeutic agent by mediating a range of stem cells.^13^ For example, the 29-mer (PEDF residues 93–121) shows therapeutic potential to heal injured muscle and tendon by activating the muscle satellite cells and CD146^+^ tendon stem/progenitor cells in their respective niches.^14,15^ Recently, a PEDF receptor (PEDFR), patatin-like phospholipase domain containing 2 (PNPLA2, also termed adipose triglyceride lipase; ATGL), has been found to be critical for linking the 29-mer bioactivity to suppress inflammatory responses in corneal epithelial cells.^16^ In addition, PEDF 44-mer (residues 78–121) can induce the proliferation of limbal stem cells (LSCs) through PNPLA2/signal transducer and activator of transcription 3 (STAT3) signalling.^17^ Meanwhile, STAT3 phosphorylation induced by the 44-mer in LSCs is blocked by atglistatin, a PEDFR-specific inhibitor.^17^ Although PEDF peptide is shown to promote regeneration of muscle, tendon, and cornea, the potential effect of the 29-mer on chondrogenesis of stem cells is unclear.

Here, the effects of 29-mer on the proliferation and chondrogenic differentiation of BM-MSCs were analyzed by in vitro experiments. A rat knee OA model by intra-articular injection of monosodium iodoacetate (MIA) was employed to detect the effects of 29-mer/hyaluronic acid (HA) on the regeneration of chondrocytes and cartilage matrix. Mechanism exploration was performed by detecting the effects of PEDFR and STAT3 inhibitors on the therapeutic effect of 29-mer/HA on rat OA.

## Methods

### Materials

Hyaluronic acid sodium salt (from *Streptococcus equi* bacterial glycosaminoglycan polysaccharide), 5-bromo-2'-deoxyuridine (BrdU), and Alcian blue 8 GX were purchased from MilliporeSigma (USA). Anti-BrdU, anti-aggrecan, and anti-Sox9 were purchased from GeneTex (Taiwan). Anti-phospho-Stat3 (Tyr705) was purchased from Cell Signaling Technology (USA). PE-conjugated anti-rat CD90 antibody was purchased from Abcam (USA). Atglistatin, SC-1, and cryptotanshinone (CPT) were purchased from Selleckchem (USA). Also, 29-mer and a control peptide (PEDF Glu97-Ser114) were modified to increase stability by acetylation at the NH_2_ terminus and amidation at the COOH terminus and synthesized to the purity > 95%, determined using mass spectrometry by the vender (GenScript, USA).

### Animal studies

All animals were housed in an animal room under temperature control (24°C to 25°C) and a 12:12 light-dark cycle. Standard laboratory chow and tap water were available ad libitum. Animals in research were treated in compliance with the ARRIVE guidelines, and an ARRIVE checklist is included in the Supplementary Material to show that the guidelines were adhered to in this study. A total of 204 adult ten-week-old male Sprague-Dawley rats (mean initial body wt = 312 g (standard deviation (SD) 11)) were supplied by BioLASCO (Taiwan).

### Induction of OA in rats and 29-mer treatment

Rats were anaesthetized by an intraperitoneal injection of xylazine (10 mg/kg) and then the right knees were injected with MIA (1 mg dissolved in 25 µl of sterile saline), using a 27-gauge needle through the patellar ligament of the knees.^18,19^ The left knee joints (control) were injected with saline. To prepare the 29-mer/HA, the 29-mer was dissolved in dimethylsulfoxide (DMSO) to make a 10 mM stock solution. Equal volumes of DMSO and 29-mer (0.5 µl) were dissolved in HA. At days 8 and 12 after MIA injection, the right knees (n = 6 per group) were randomly injected by: 1) 29-mer/25 µl of 5% HA (final concentration of 29-mer at 0.2 mM); 2) DMSO vehicle/5% HA; 3) control peptide/5% HA; 4) 5% HA alone; and 5) 29-mer/25 µl of phosphate-buffered saline (PBS; final concentration of 29-mer at 0.2 mM). For inhibitor studies, atglistatin, SC-1, and CPT were dissolved in 25 µl of 5% HA (final concentrations at 1 mM) and were intra-articularly injected into right knees for 60 min, before the 29-mer/HA injection at days 8 and 12 after MIA injection.

### Macroscopic observation and histopathology

The knee joints were harvested after MIA injection for 14 days. The femoral condyle and tibial plateau were carefully separated and then rinsed with PBS and painted with India ink (BBL; Becton Dickinson, USA). The excess ink was removed by washing with PBS. The cartilage degeneration and bone destruction of the femoral condyle and tibial plateau were graded using a macroscopic score on a scale of 0 to 5 points as previously described.^20^

### Histological examination of the knee joint

The knee joints were dissected and decalcified with Shandon TBD-2 decalcifier (Thermo Fisher Scientific, USA) at room temperature (RT) for two days, and then fixed in a 4% paraformaldehyde (PFA) for six hours. Subsequently, the joints were embedded in paraffin blocks and mid-sagittally sectioned at 5 µm. To examine the severity of synovitis, the whole knee joints were harvested at two weeks after MIA injection and then the sections were stained with haematoxylin and eosin (H&E). The infrapatellar fat pad (IFP) inflammation was scored on a scale of 0 to 6 points, and was semi-quantitatively evaluated as previously described.^20^

To assess the severity of cartilage degeneration, sections were stained with Safranin O/Fast green and Col2 using a cartilage staining kit (#MK310; TaKaRa, Japan). The levels of matrix staining were scored on a scale of 0 to 4 as previously described.^21^ The percentage of Col2-positive area per AC surface was evaluated from the photographs by using a computer-assisted image analyzer (Adobe Photoshop CS3 10.0; Adobe, USA).

Osteoarthritic changes at medial tibial plateau at day 25 after MIA injection were graded using the Osteoarthritis Research Society International (OARSI) histopathological scoring system on a scale of 0 to 24 points as previously described.^20,22^ To determine the IFP inflammation, OARSI, and matrix staining scores, ten sections per knee joint were carefully prepared to include the most severely degenerated area and photographed using a Nikon Eclipse 80i microscope (Nikon, Japan). The mean score of each group was estimated by two observers (TCH, SIY) in a blinded manner based on 60 sections.

### In vivo detection of DNA synthesis and immunofluorescence staining

BrdU was reconstituted in DMSO as stock (80 mM). Then, 150 μl of BrdU mixed with 350 μl of PBS was intraperitoneally injected into rat, immediately after intra-articular injection of the 29-mer/HA at days 8 and 12 after MIA injection. Deparaffinized sections were treated with 1N HCl at RT for 1 h, and DNA synthesis was assessed by anti-BrdU antibody. Next, tissue sections were blocked with 10% goat serum and 5% BSA for one hour. Immunostaining was carried out using primary antibodies against aggrecan, Sox9, and BrdU (all 1:100 dilution) at 37°C for two hours, followed by incubation with the appropriate rhodamine- or FITC-conjugated donkey IgG for one hour at RT. Images were captured using a Zeiss epifluorescence microscope with a CCD camera (Zeiss, Germany), and blinded quantification was performed in triplicate by manually counting within each section. Anti-BrdU antibody was also recognized by peroxidase-labelled goat immunoglobulin (1:500 dilution) for 20 minutes and then incubated with chromogen substrate (3,3'-diaminobenzidine) for two minutes before counterstaining with haematoxylin.

### Pharmacokinetic analysis of the 29-mer/HA in AC

Next, 29-mer (1 mg) dissolved in 25 μl of HA was intra-articularly injected into normal rat knees, at the following timepoints: 0.5, one, two, three, and four hours (n = 3 per timepoint). Immediately after rat euthanasia, synovial fluid was collected by use a 27G needle and syringe to flush sterile saline three times (100 µl each time) into the knee joint cavity, pooled into a 1.5 ml centrifuge tube, and centrifuged at 12,000× g for one minute. The supernatant was stored at -80°C until use. The AC was homogenized in CelLytic MT Mammalian Tissue Lysis/Extraction Reagent (MilliporeSigma), and the lysed samples were centrifuged for ten minutes at 12,000× g. The supernatant was stored with protease inhibitor cocktail (MilliporeSigma) and the levels of 29-mer were measured using liquid chromatography–mass spectrometry (LC-MS) as previously described.^23^

### Assessment of changes in hind paws weight distribution

At day 28 post-MIA injection, the changes between the right (osteoarthritic) and left (contralateral control) limbs were detected by an incapacitance tester (PM-01; SINGA, Taiwan) and used as an index of joint discomfort. The rats (n = 6 per group) were carefully placed in a measuring chamber, and the weightbearing force exerted on the hind limbs was averaged over a ten-second period and readings for two minutes. The weight distribution ratio was calculated by the following equation: (1-(mean ∆ weight of treated group/mean ∆ weight of contralateral control))×(100).^19^

### Isolation and culture of MSCs

Sprague-Dawley rats were anaesthetized and their femora were aseptically harvested, washed in a mixture of PBS and 1% penicillin/streptomycin for five minutes, dissected of all soft-tissue, transected at their epiphysis, and their marrow cavity rinsed repeatedly with a mixture of heparin (AGGLUTEX INJ 5,000 U/ml; working conc. 100 U/ml) and Dulbecco’s Modified Eagle Medium (DMEM; Gibco, Thermo Fisher Scientific). The harvested cells were collected, dispersed by pipetting, and centrifuged at 1,000× g for five minutes at RT. Cell pellets were resuspended with DMEM and then the cell suspension was transferred to a 15 ml centrifuge tube containing 5 ml of Percoll (1.073 g/ml; MilliporeSigma). After centrifugation at 1,500× g for 30 minutes, the mononuclear cells in the middle layer were obtained, washed three times with PBS, and suspended in expansion medium (low-glucose DMEM with 10% heat-inactivated FBS, 1% penicillin/streptomycin). The surface antigen of rat BM-MSCs determined by flow cytometry showed strongly positive for CD90 (> 98%). BrdU immunostaining of BM-MSCs was performed as in Ho et al’s^14^ study.

### Chondrogenic differentiation of BM-MSCs

The procedure was performed as previously described.^24^ Briefly, 5 × 10^3^ expanded BM-MSCs were placed in each well of a 96-well plate and exposed to 150 µl of chondrogenic medium (high-glucose DMEM with 100 nM dexamethasone, 0.17 mM ascorbic acid-2 phosphate, 0.35 mM proline, 10 µg/ml of insulin, 5 µg/ml of transferrin, 5 ng/ml selenium, 1 mM sodium pyruvate, 2 mM L-glutamine, and 2% FBS) supplemented with 10 ng/ml transforming growth factor (TGF)-ß3 (R&D Systems) and 10 µM 29-mer by feeding three times a week.

### Alcian blue staining and quantification

BM-MSCs were cultured in chondrogenic-inducing medium for three weeks. For Alcian blue staining, culture dishes were rinsed twice with PBS, fixed in 4% PFA for 15 minutes, and incubated in 1% (w/v) Alcian blue 8 GX in 0.1N HCl overnight as previously described.^25^ For semi-quantitative analysis, Alcian blue-stained cultures were extracted with 6M guanidine HCl for six hours at RT. The absorption of the extracted dye was measured at 650 nm in a SpectraMax Gemini microplate reader (Molecular Devices; USA).

### Quantitative real-time PCR

Total RNA was extracted from the cells or cartilage of rat knees using TRIzol (Life Technologies, USA) and quantitative real-time polymerase chain reaction (PCR) were performed as in Ho et al’s^15^ study. Primers used in the experiment are listed in Table I. The expression of glyceraldehyde 3-phosphate dehydrogenase (*GAPDH*) gene, which showed no significant difference between the experimental groups, was set as the internal control gene. All determinations were made in triplicate. The Ct value of the PCR product of interest and *GAPDH* control messenger RNA (mRNA) were then used to calculate the relative quantities of mRNA.

**Table I.** Primers used for quantitative real-time polymerase chain reaction.

Target gene	Primer (sense)	Primer (antisense)	Accession no.
rat aggrecan	5`-TTGGAAATCCAGAACCTTCG	5`-GTCCAGTGTGTAGCGTGTGG	J03485
rat COL2a1	5`-GGAAGAGCGGAGACTACTGG	5`-TTGCAGAAGACTTTCATGGC	XM_006242308
rat Sox9	5`‐CCGGATCTGAAGAAGGAGAG	5`‐CTTGACGTGTGGCTTGTTCT	NM_080403
rat GAPDH	5`‐AGACAGCCGCATCTTCTTGT	5`‐CTTGCCGTGGGTAGAGTCAT	X02231.1

COL2a1, collagen type 2 alpha 1; GAPDH, glyceraldehyde 3-phosphate dehydrogenase; Sox9, SRY-type high mobility group box 9.

### Western blot analysis

Cell lysis and SDS–PAGE were performed as previously described.^15^ The band intensities in immunoblots were measured with a Model GS-700 imaging densitometer (Bio-Rad Laboratories, USA) and analyzed using Labworks 4.0 software (Labworks, USA).

### Statistical analysis

The significance of the difference between groups was analyzed with the independent-samples *t*-test. Differences between groups were examined for statistical significance using one-way analysis of variance (ANOVA), followed by Dunnett’s post hoc test. The results are expressed as mean and standard error of the mean (SEM). p < 0.05 was considered to be statistically significant.

## Results

### The 29-mer promotes the proliferation and chondrogenic differentiation of BM-MSCs cultured in different defined media

**Fig. 1:**
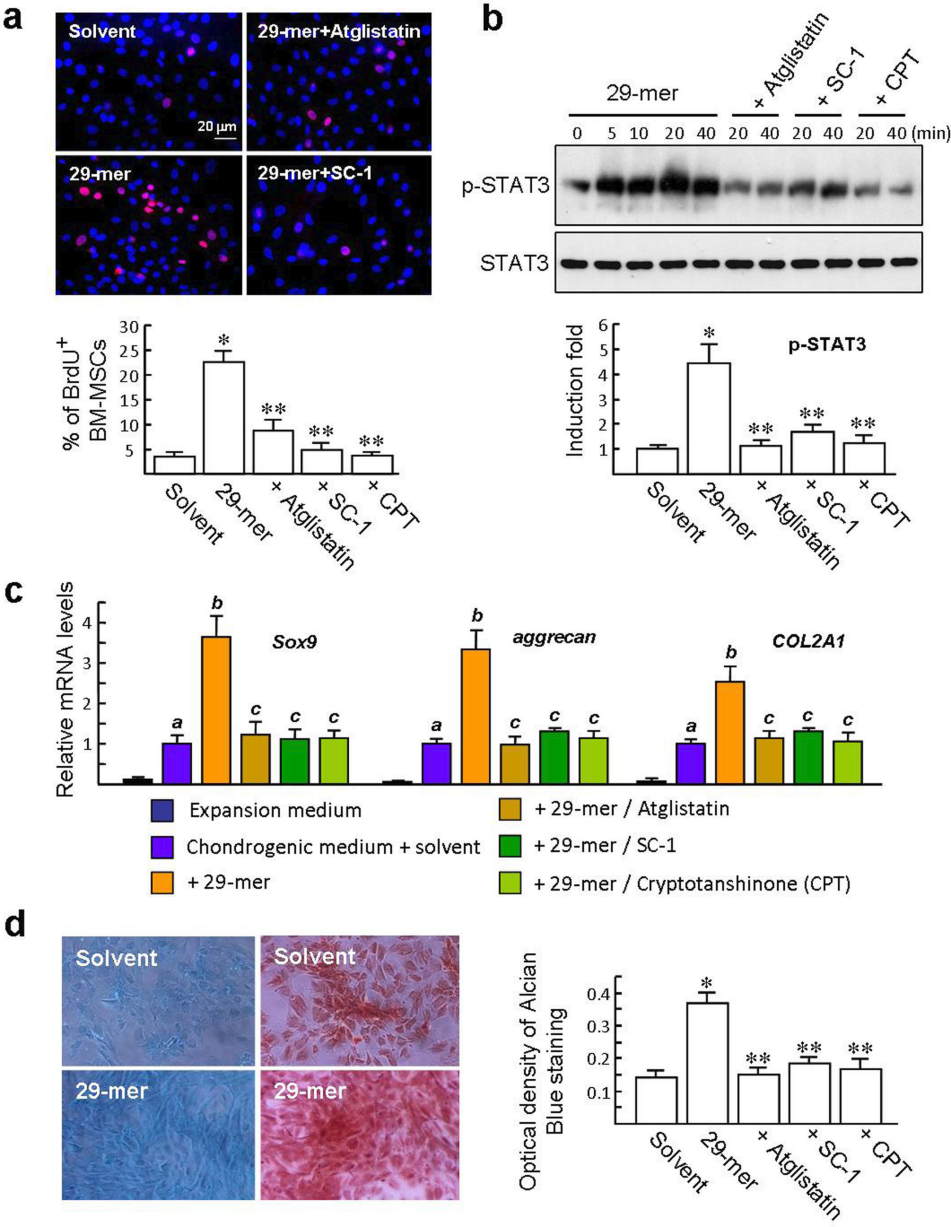
The proliferation and chondrogenic differentiation of bone marrow mesenchymal stem/stromal cells (BM-MSCs) are regulated by the 29-mer via pigment epithelium-derived factor receptor (PEDFR)/signal transducer and activator of transcription 3 (STAT3) signalling. a) Immunostaining of 5-bromo-2'-deoxyuridine (BrdU) incorporation in BM-MSCs cultured in expansion medium (n = 6). *p < 0.001 vs solvent treatment. **p < 0.001 vs 29-mer treatment. b) Representative immunoblots and densitometric analysis of p-STAT3 (Tyr 705) induced by the 29-mer and 29-mer/inhibitor for 20 minutes. *p < 0.001 vs solvent. **p < 0.003 versus 29-mer. c) BM-MSCs were cultured in chondrogenic medium with the 29-mer for seven days prior to quantitative polymerase chain reaction (qPCR) analysis of the expression of chondrogenic genes (n = 6). *^a^*p < 0.001 versus MSCs cultured in expansion medium. *^b^*p < 0.001 versus BM-MSCs cultured in solvent/chondrogenic medium. *^c^*p < 0.007 vs 29-mer-treated BM-MSCs. d) Representative micrographs of Alcian blue- and Safranin O-stained sections from BM-MSCs cultured at chondrogenic medium for three weeks. Optical density (OD) values of Alcian blue are shown relative to the total protein determined by bicinchoninic acid (BCA) assay. *p < 0.001 versus solvent. **p < 0.003 versus 29-mer. All p-values in this figure were calculated using one-way analysis of variance (ANOVA). COL2a1, collagen type 2 alpha 1; CPT, cryptotanshinone.

BM**-**MSCs were expanded for seven days in culture and then exposed to the 29-mer (10 μM) for 24 hours. Cell proliferation was detected by BrdU pulse-labelling for four hours and analyzed by BrdU immunostaining (red colour; Figure 1a). The 29-mer treatment increased BrdU^+^ levels by 6.6-fold, compared with solvent treatment. The assay also revealed that pretreatment with inhibitors targeting PEDFR (atglistatin) and STAT3 (SC-1, CPT) significantly suppressed the 29-mer effect (p < 0.001, one-way ANOVA). Meanwhile, western blotting demonstrated that the 29-mer can induce phosphorylation of STAT3 in BM-MSCs, occurring five to 40 minutes after the treatment, but the effect was abolished by such inhibitors (Figure 1b).

Next, the expanded BM-MSCs were cultured in a chondrogenic defined medium and stimulated with the 29-mer for one week. Quantitative real-time PCR analysis revealed that the 29-mer induced increases of chondrogenic genes, including *Sox9*, *aggrecan*, and *Col2a1* expressions by 3.6-, 3.3-, and 2.5-fold, compared to BM-MSCs treated with solvent. Meanwhile, inhibitors targeting PEDFR and STAT3 signalling reduced the 29-mer-induced gene expressions to near basal levels (Figure 1c). After the 29-mer treatment for 21 days, the biosynthesis of cartilage matrix in culture were stained by Alcian blue and showed a 2.6-fold increase in the levels of glycosaminoglycans (GAGs), compared to the solvent control (Figure 1d). Likewise, Safranin O staining also showed higher levels of proteoglycans induced by the 29-mer. The biosynthesis of GAGs induced by the 29-mer was also abolished by inhibitors. Collectively, these findings indicate that the 29-mer induces the activation of PEDFR/STAT3 signalling to promote the expansion and chondrogenic differentiation of BM-MSCs cultured in different conditions.

### The 29-mer/HA prevents AC destruction induced by MIA

**Fig. 2:**
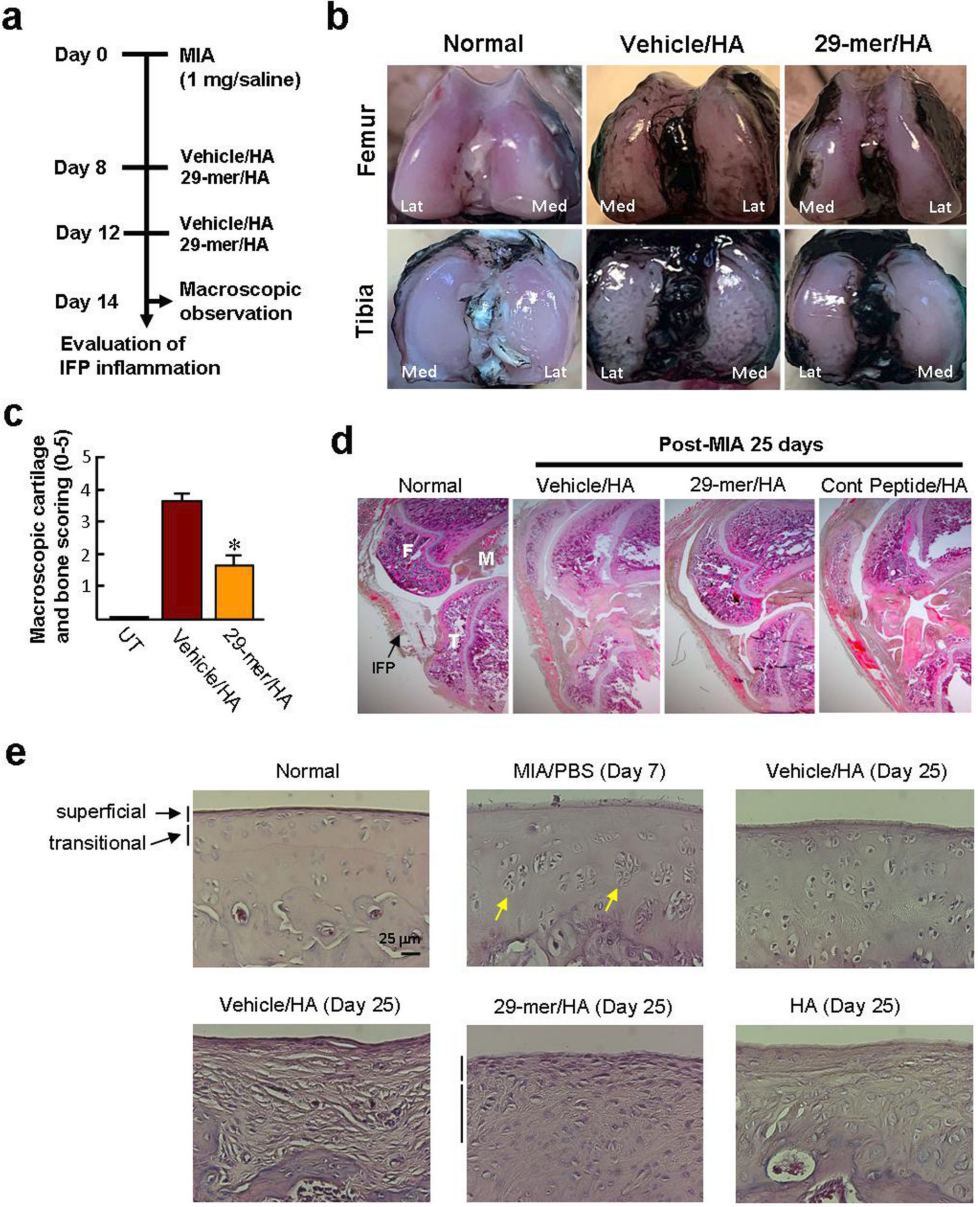
The 29-mer/hyaluronic acid (HA) is effective in preventing articular cartilage (AC) destruction induced by monosodium iodoacetate (MIA). a) Experimental study schema. The right knee joint had an intra-articular injection of MIA at day 0. Subsequently, the right knee joint was intra-articularly injected with the 29-mer/HA at days 8 and 12 after MIA injection. b) Representative macroscopic images of the surface of femoral and tibial condyles stained with India ink (n = 3 per group). Lat, lateral; Med, medial. c) Macroscopic cartilage score. *p < 0.001 versus vehicle/HA group. d) and e) Haematoxylin and eosin (H&E) staining of longitudinal knee joint sections. Representative graphs from two independent experiments (n = 6 per group) are shown (original magnification, ×40 and ×400). All p-values in this figure were calculated using one-way analysis of variance. Arrows: chondrocyte degeneration with prominent pyknotic nuclei. F, femoral condyle; T, tibial condyle; M, meniscus; IFP, infrapatellar fat pad; UT, untreated.

A schematic timeline describing the rats receiving different treatments and knee joints harvested for histological analyses at day 14 after MIA injection is shown in Figure 2a. Generally, the AC surfaces of tibia and femur in the vehicle/HA group were irregular and showed multiple fibrotic depositions after staining with India ink (Figure 2b). In contrast, representative AC surface of the femoral condyle and tibial plateau in the 29-mer/HA group was smoother, indicating milder damage on the AC surfaces compared to the vehicle/HA group (score 1.7 vs 3.7; Figure 2c).

H&E-stained sections of the vehicle/HA and control peptide/HA groups showed loss of cartilage integrity throughout the AC area, hyperplasia of IFP tissue, and subchondral bone collapse, especially at weightbearing sites, after MIA injection for 25 days (Figure 2d). In contrast, the 29-mer/HA group showed moderate preservation of the joint space and AC surface continuity. Microscopically, MIA induced extensive chondrocyte death in AC, after injection into the femorotibial joint space of rats for seven days, similar to a previous observation.^18^ Injection of vehicle/HA at days 8 and 12 after MIA injection, and harvesting the knee joint at day 25 after MIA injection revealed loss of chondrocytes, superficial fibrillations, and scattered cell clusters at cartilage surface of the femoral condyle (Figure 2e), indicating failure to prevent the progression of OA induced by MIA. We also noted that intra-articular injection of the HA alone had no therapeutic effect on MIA-injured AC in rats, which was consistent with a previous finding.^26^ On the other hand, the femoral condyles in the 29-mer/HA group exhibited large numbers of newly generated cells filled in the AC surface, consisting of spindle cells aligned parallel to the superficial surface and accumulation of round cells at the transitional zone.

**Fig. 3:**
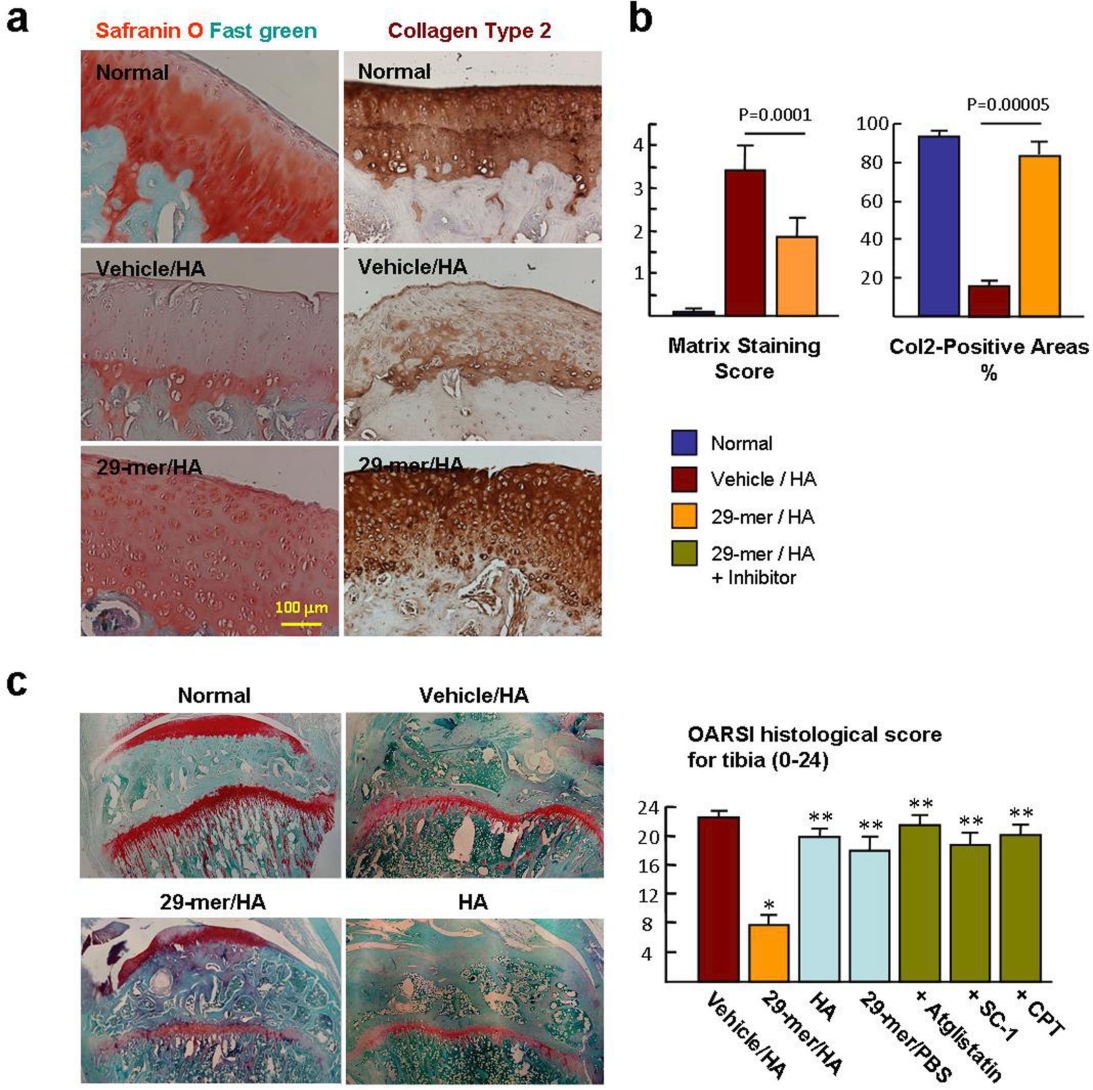
Histological analysis of articular cartilage (AC) defects. a) Representative micrographs of femoral condyles stained by Safranin O/Fast green and collagen type 2 (Col2) at day 25 post-monosodium iodoacetate (MIA) injection (n = 6 per group). Magnification, ×200. b) Evaluation of the levels of cartilage matrix on a scale of 0 to 4 points. c) Representative images of Safranin O/Fast green stained tibial condyle. Histological analyses of the tibial condyles were graded by Osteoarthritis Research Society International (OARSI) histological score at day 25 post-MIA injection (n = 6 per group). *p < 0.001 vs vehicle/hyaluronic acid (HA) group. **p < 0.001 vs 29-mer/HA group. All p-values in this figure were calculated using one-way analysis of variance. CPT, cryptotanshinone; PBS, phosphate-buffered saline.

To evaluate cartilage regeneration, the levels of cartilage matrix at the AC surface were investigated after MIA injection for 25 days. The sections of vehicle/HA group showed reductions of sulfated proteoglycan and Col2 throughout the AC region of femoral condyles, reflecting severe cartilage degeneration and failure of cartilage regeneration (Figures 3a and 3b). In contrast, the sections of 29-mer/HA group showed positive staining for Safranin O and Col2 at the AC surface, reflecting that the cartilage matrix is regenerated in the MIA-damaged AC.

Next, the OARSI grading system (score 0 to 24) was used to evaluate the OA histopathological progressions after MIA injection for 25 days. Typical Safranin O staining images of the tibial condyle in the vehicle/HA group showed extensive denudation and deformation. In contrast, the 29-mer/HA group had an intact surface, although parts of the surface exhibited vertical fissures, consistent with lower OARSI scores than the vehicle/HA group (mean score 7.8 (SEM 1.2) vs 22.4 (SEM 0.7); Figure 3c). Notably, the 29-mer/HA effect was almost completely blocked by inhibitors targeting PEDFR and STAT3, respectively. The findings imply that both PEDFR and STAT3 signalling are crucial for the 29-mer/HA to heal the AC defects induced by MIA.

### Inflammatory responses in the infrapatellar fat pad induced by MIA are alleviated by treatment with the 29-mer/HA

**Fig. 4:**
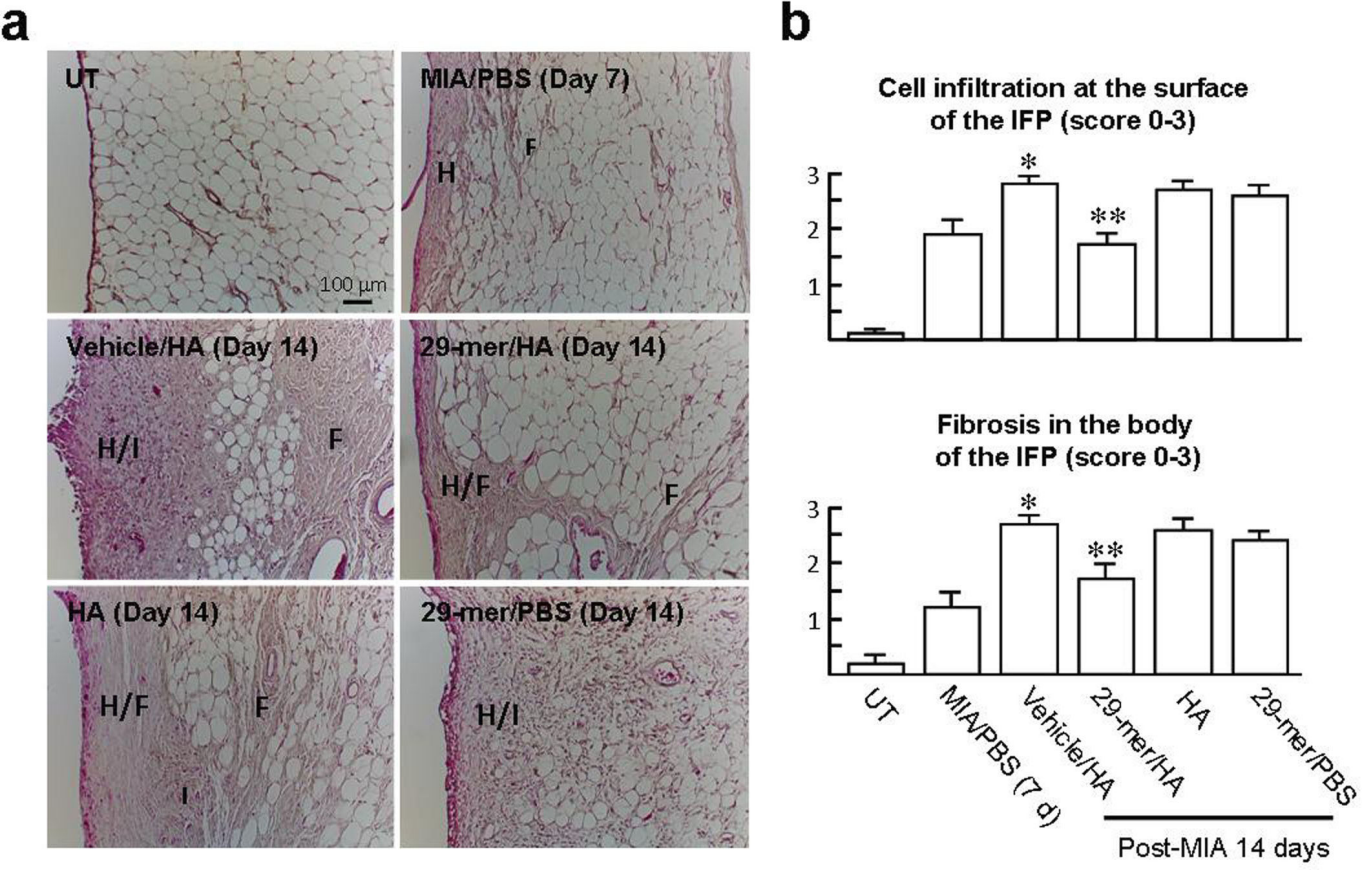
The 29-mer/hyaluronic acid (HA) blocks the development of infrapatellar fat pad (IFP) inflammation induced by monosodium iodoacetate (MIA). a) Representative histological images of haematoxylin and eosin (H&E)-stained IFP in different experimental groups after MIA injection for seven and 14 days. b) IFP inflammation score was graded by the severity of cell infiltration and fibrosis in the IFP (n = 3 per group). All p-values in this figure were calculated using one-way analysis of variance. H, cellular hyperplastic response; I, cell infiltration; F, fibrotic deposits. *p < 0.001 vs MIA/PBS group. **p < 0.01 vs vehicle/HA group. PBS, phosphate-buffered saline; UT, untreated.

The 29-mer/HA group generally showed milder structural changes in the IFP, compared to the vehicle/HA group (Figure 2d), suggesting that a mild inflammation is induced. To clarify, an IFP inflammation score system was used to evaluate synovitis in experimental groups, due to the scoring method comparable to the MIA-induced arthritis in rats that has been documented.^20^ As shown in Figure 4, intra-articular injection of MIA for seven days caused IFP inflammation, as indicated by the presence of cell infiltration and cellular hyperplasia at the surface of the IFP (mean score 1.9 (SEM 0.2)) and the fibrosis in the body of the IFP (mean score 1.2 (SEM 0.3)). At day 14 post-MIA injection, the vehicle/HA group showed more extensive cell infiltration and fibrosis occupied in IFP (mean score 2.8 (SEM 0.1) and 2.7 (SEM 0.2), respectively). In contrast, the 29-mer/HA group showed lower cell infiltration and fibrosis (mean score 1.7 (SEM 0.2) and 1.7 (SEM 0.3), respectively) than the vehicle/HA group. However, treatment with HA alone or the 29-mer/PBS was ineffective in these regards. The results suggest that the 29-mer/HA is able to alleviate the MIA-induced IFP inflammation.

### Pharmacokinetic analysis of the 29-mer in cartilage tissue

**Fig. 5:**
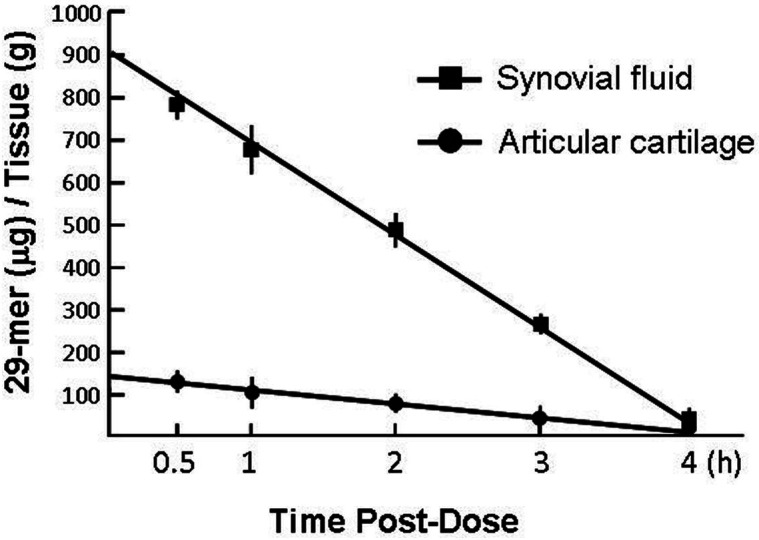
Liquid chromatography–mass spectrometry analysis of the distribution of intra-articularly injected 29-mer. Each set of data points between 0.5 hours and four hours was fit to a regression line to extrapolate an initial maximum level of the 29-mer in each tissue. T_1/2_, elimination half-life. n = 3 per timepoint.

The pharmacokinetic behaviour of the 29-mer after intra-articular injection was measured using LC-MS and showed distribution at the AC and synovial fluid with T_1/2_ (half-lives) of 1.82 hours and 2.07 hours, respectively (Figure 5). By two hours, the 29-mer levels in synovial fluid stayed about five-fold higher than in the AC. Notably, the 29-mer rapidly decreased in a time-dependent manner; it remained in AC and synovial fluid for only four hours after 29-mer/HA injection.

### The 29-mer/HA ameliorates the MIA-induced joint discomfort

**Fig. 6:**
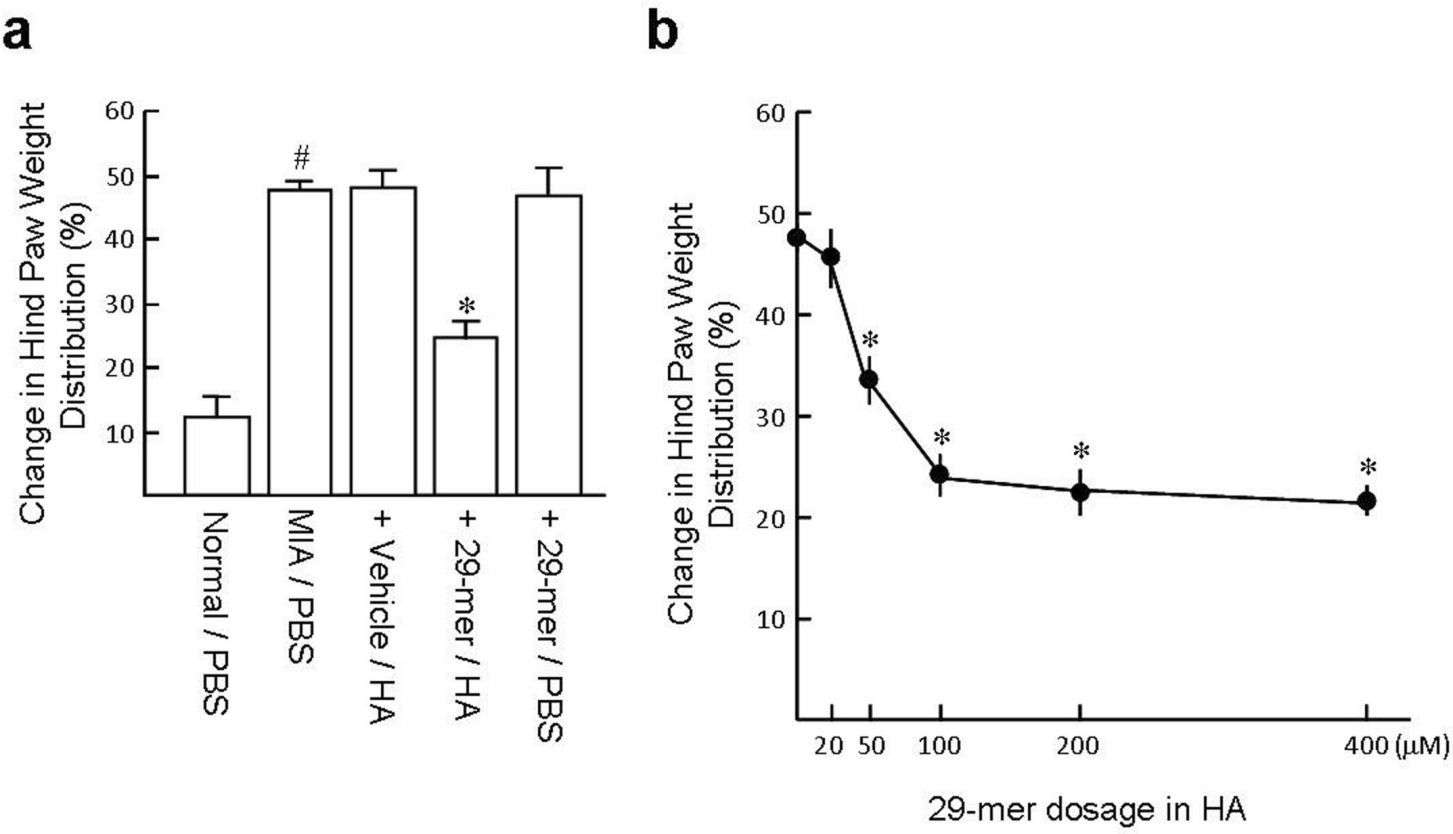
Change in hind paw weight distribution (weightbearing) was assessed at day 28 post-monosodium iodoacetate (MIA) injection. a) Comparison of antinociceptive effect of 29-mer infused with hyaluronic acid (HA) and phosphate-buffered saline (PBS). b) Dosage effect (n = 6 per group). The right knee joint injected with PBS was set as the normal/PBS control. ^#^p < 0.001 vs normal/PBS. *p < 0.01 versus vesicle/HA. All p-values in this figure were calculated using one-way analysis of variance.

At day 28 post-MIA injection, analysis of the weightbearing shift by incapacitance tester showed that the vehicle/HA group increased in weightbearing shift compared to the normal/PBS group (47.6% vs 12.1%; Figure 6a). Treatment with a range of doses of the 29-mer (50 to 400 μM in HA) demonstrated better weightbearing ability than the vehicle/HA group (33.9% to 21.6%; Figure 6b). In contrast, the 29-mer/PBS treatment had no significant antinociceptive effect.

### The 29-mer/HA stimulates chondrocyte regeneration in the damaged AC

**Fig. 7:**
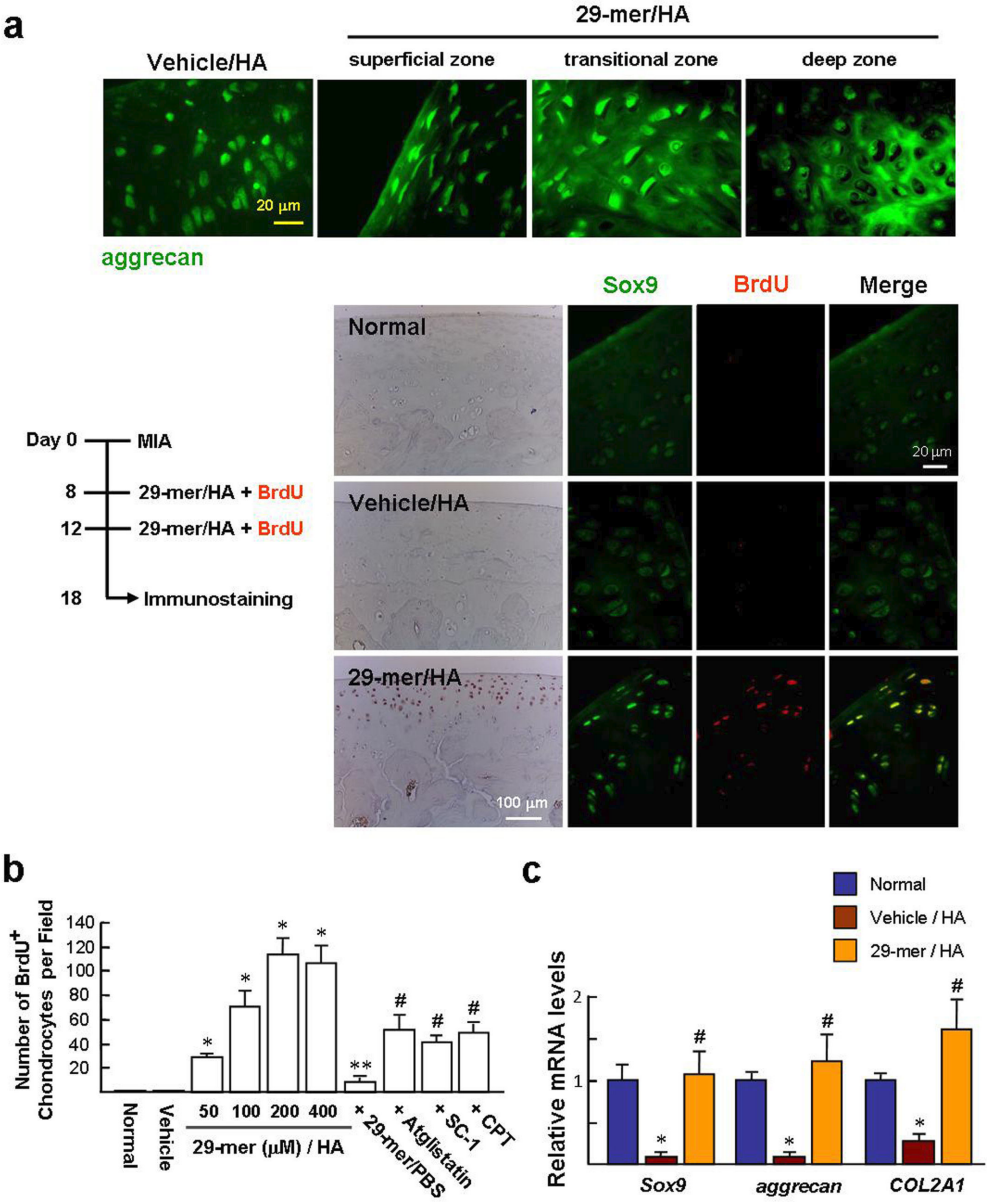
Analysis of the phenotype of newly generated cells in monosodium iodoacetate (MIA)-injured articular cartilage (AC) after the 29-mer/hyaluronic acid (HA) treatment. a) Immunostaining of aggrecan, 5-bromo-2'-deoxyuridine (BrdU), and SRY-type high-mobility group box 9 (Sox9). Representative images are from six sections per rat knee joint (n = 6). b) Analysis of the levels of BrdU-positive cells at damaged AC by a double-blind on an average of six randomly selected fields per section (field: 200× magnification). *p < 0.001 vs vehicle group. **p < 0.001 vs 200 µM 29-mer/HA group. ^#^p < 0.005 vs 200 µM 29-mer/HA. c) Chondrogenic gene expressions in damaged AC at day 18 post-MIA injection. The results are normalized to glyceraldehyde 3-phosphate dehydrogenase (*GAPDH*) (n = 4 per group). *p < 0.001 versus normal group. ^#^p < 0.001 vs vehicle/HA. All p-values in this figure were calculated using one-way analysis of variance. COL2A1, collagen type 2 alpha 1; CPT, cryptotanshinone; mRNA, messenger RNA; PBS, phosphate-buffered saline.

To detect the phenotype of newly generated cells in the MIA-damaged AC, after treatment with the 29-mer/HA, immunofluorescence staining of aggrecan (the major proteoglycan generated from chondrocytes) showed almost all cells stained positive for aggrecan (Figure 7a). Of note, the vehicle/HA group had only a pale aggrecan staining. Alternately, the BrdU injection was given simultaneously with the 29-mer/HA treatment (days 8 and 12). Large numbers of BrdU-positive cells were seen in the injured AC. Dual-immunofluorescence staining revealed that almost the entire BrdU-positive cells stained positive for Sox9. In contrast, the AC region of the vehicle/HA group was stained negative for BrdU, reflecting that there is no spontaneous chondrocyte regeneration after MIA injection. The levels of BrdU-labelled chondrocytes induced by the 29-mer (50 to 400 µM dissolved in HA) were increased in a dose-dependent manner (Figure 7b; mean 28 (SEM 3.1) to 107 (SEM 13.7) cells per field). Also, the 29-mer/HA effect was blocked by atglistatin, SC-1, and CPT, respectively. Furthermore, the surface tissue from the injured AC treated with the 29-mer/HA showed the gene expression levels of *Sox9*, *aggrecan*, and *Col2a1* to be similar to those expressed in normal AC tissue (Figure 7c), supporting the regeneration of chondrocytes induced by the 29-mer/HA.

## Discussion

In this study, OA induced by MIA in rats featured extensive chondrocyte degeneration/death in AC, leading to the replacement of hyaline cartilage by fibrocartilaginous tissue, with no cartilage regeneration. The OA model reflects the poor self-healing ability of cartilage that results in the medical burden of human OA. We have demonstrated for the first time that the 29-mer/HA displays a therapeutic effect on the damaged AC by the regeneration of cartilage. The increased expression of Col2 and aggrecan shows the features of hyaline cartilage in the regenerated area. Additionally, we showed that the 29-mer can augment the proliferation and chondrogenic potential of BM-MSCs in culture. The 29-mer bioactivity on MSCs may be a potential mechanism in promoting cartilage repair in vivo.

In the present study, the sources of stem cells involved in the AC repair remain unclear. Stem cells have been found to reside in several tissues of the knee joint. For example, the superficial zone of AC contains MSC-derived chondrocyte progenitors.^27,28^ The adult synovium in the joint cavity reportedly has cell populations with the functional behaviour of MSCs.^9^ CD105- and CD166-positive MSCs induced by a static magnetic field have been found to be recruited to heal the cartilage damage.^27^ Given these findings, it is conceivable that MSCs may contribute to the proliferative burst of chondrogenic cells in the damaged AC in response to the 29-mer/HA treatment.

In the present study, we demonstrated that BM-MSCs exposed to the 29-mer in culture led to an increase in the expression of chondrogenic genes and biosynthesis of cartilage matrix. These results suggest a new method to promote the chondrogenic potential of BM-MSCs. Indeed, the chondrogenic potential of BM-MSCs is the theoretical basis for microfracture surgery that creates holes in the subchondral bone to facilitate BM-MSC mobilization to the AC surface to heal cartilage in clinical treatment of small symptomatic AC lesions.^29^ However, a mixed fibrocartilage tissue is commonly found to occupy the AC defects rather than hyaline cartilage.^30^ Whether the 29-mer/HA can benefit the microfracture surgery outcome by promoting the regeneration of hyaline cartilage is interesting and awaits further investigation.

In this study, the 29-mer peptide was dissolved in DMSO to make a high-concentration stock solution in order to reduce the dilution of 29-mer in the 29-mer/HA mixture. Our presented data do not address whether the DMSO vehicle has an inhibitory effect on HA-induced cartilage regeneration, rendering MIA-induced OA unresponsive to HA treatment. However, we noted that, similar to the vehicle/HA, HA alone was still incapable of reducing AC damage and IFP inflammation induced by MIA. These results suggest that the DMSO vehicle does not interfere with cartilage regeneration. However, we found that the HA was necessary for the 29-mer to exhibit a therapeutic effect in rat OA. The exact mechanism of HA in this study remains elusive. HA is a common component of sustained-release formulation and may increase the retention of the 29-mer in the knee joint cavity. Alternatively, it is known that association of HA with the surface CD44 glycoprotein of MSCs facilitates the recruitment of MSCs into wound tissues.^31^ Therefore, it is also possible that HA acts in binding the MSCs to facilitate their mobilization into the injured AC.

In the present study, we have shown that the PEDFR and STAT3 signalling pathways are essentially involved in the proliferation of BM-MSCs induced by the 29-mer, supporting previous findings that the pathway involves the proliferation of LSCs and satellite cells induced by the PEDF and 29-mer.^14,17^ Interestingly, chondrogenic medium supplemented with the 29-mer improves the chondrogenic differentiation of BM-MSCs and the effect is also blocked by STAT3 inhibitors. Our findings support the previous notion that STAT3 signalling can stimulate *Sox9* gene expression in chondrocytes and human BM-MSCs.^32,33^ TGF-β is known to mediate chondrogenic differentiation of MSCs, through activating Smad signalling to augment the gene expression of *Sox9*, as a critical component of chondrogenic medium.^34^ SOX9 is a master chondrogenic transcription factor responsible for upregulation of *COL2A1* and *aggrecan* gene expression. Subsequently, aggrecan can covalently link sulphated GAGs, to confer an osmotic swelling property of cartilage.^6,7^ One drawback of MSCs is that they tend to differentiate into hypertrophic chondrocytes, reducing their ability to cope with pressure and shear force.^5,35^ Our findings suggest that crosstalk between the PEDF/STAT3 and TGF-β/Smad signalling pathways may help to synthesize hyaline cartilage in MSCs.

Limitation of mobility induced by joint pain is the major complaint of OA patients. Thus, pain reduction is one of the primary targets of therapy in OA. The source of pain is multiple. Cartilage destruction, such as dislodged cartilage, may induce pain through peripheral afferent and dorsal root ganglion (DRG) neurones.^36^ In this study, the effect of 29-mer/HA on blockage of AC destruction is supported by reduction of the imbalanced hind limb weight distribution induced by MIA, implying relief of the joint pain in animals. Intra-articular injection of MIA is known to cause the axonal injury of DRG cells.^37^ Interestingly, the PEDF 44-mer, a precursor of the 29-mer, has been demonstrated to be able to promote functional regeneration of damaged corneal nerves induced by an experimental surgery.^38^ The potential value in treating OA pain warrants further investigation.

It has been demonstrated that the intra-articular injection of MIA (1 mg) induces an acute inflammation in synovial membrane, followed by AC degradation and bone disruption in rats.^20^ MIA injection in the animal knee is considered as a useful preclinical model of OA pain.^20^ Importantly, recent reports indicate that attenuation of early phase inflammation induced by MIA can prevent the development of OA pain and nerve damage in rats.^39,40^ The 29-mer/HA treatment after MIA injection can effectively block the progression of inflammation in IFP. The results suggest that the anti-inflammatory effect of the 29-mer/HA may contribute to relieving OA pain. A recent study also demonstrated the anti-inflammatory effect of the 29-mer in experimental dry eye animals.^16^ Reported recently, PEDF bears multiple functional fragments that may be detrimental to preserving AC matrix and treating arthritis in animals.^41,42^ However, by using a PEDF small fragment, the benefit of 29-mer can be shown without the unintended effects.

Our observations suggest the potential of 29-mer/HA in various clinical situations. It can be applied immediately after AC damage to prevent chondrocyte loss and cartilage destruction thereafter. It can also be used as an auxiliary treatment for cartilage surgery to improve surgical outcomes. The anti-inflammatory function of 29-mer/HA suggests that it may be used to treat OA patients, either to stimulate cartilage regeneration or to relieve the symptoms of OA. Depending on the repair potential of stem cells, it may stimulate cartilage regeneration in the early stage of OA or even in the late stage. The results of this study show that direct joint injection is effective and joint injection is a method familiar to contemporary clinicians. Long-term treatment effects and the utility of repeated injections remain to be determined.

In conclusion, structural lesions confined to the AC layer do not heal spontaneously and a number of therapeutic strategies have been used to improve AC healing. This study shows that the 29-mer/HA has a therapeutic effect on rat OA by inducing chondrogenesis in the damaged AC and inhibiting the formation of fibrocartilage, and therefore provides a potential therapeutic strategy for developing OA therapy.

## Data Availability

The data that support the finding for this study are available to other researchers from the corresponding author upon reasonable request.
